# Machine learning algorithms to predict treatment success for patients with pulmonary tuberculosis

**DOI:** 10.1371/journal.pone.0309151

**Published:** 2024-10-16

**Authors:** Shaik Ahamed Fayaz, Lakshmanan Babu, Loganathan Paridayal, Mahalingam Vasantha, Palaniyandi Paramasivam, Karuppasamy Sundarakumar, Chinnaiyan Ponnuraja

**Affiliations:** 1 Department of Statistics, ICMR ‐ National Institute for Research in Tuberculosis, Chennai, India; 2 University of Madras, Chennai, India; 3 Cognizant Technology Solutions, Chennai, India; 4 Department of Statistics, Madras Christian College, Chennai, India; Rutgers Biomedical and Health Sciences, UNITED STATES OF AMERICA

## Abstract

Despite advancements in detection and treatment, tuberculosis (TB), an infectious illness caused by the Mycobacterium TB bacteria, continues to pose a serious threat to world health. The TB diagnosis phase includes a patient’s medical history, physical examination, chest X-rays, and laboratory procedures, such as molecular testing and sputum culture. In artificial intelligence (AI), machine learning (ML) is an advanced study of statistical algorithms that can learn from historical data and generalize the results to unseen data. There are not many studies done on the ML algorithm that enables the prediction of treatment success for patients with pulmonary TB (PTB). The objective of this study is to identify an effective and predictive ML algorithm to evaluate the detection of treatment success in PTB patients and to compare the predictive performance of the ML models. In this retrospective study, a total of 1236 PTB patients who were given treatment under a randomized controlled clinical trial at the ICMR-National Institute for Research in Tuberculosis, Chennai, India were considered for data analysis. The multiple ML models were developed and tested to identify the best algorithm to predict the sputum culture conversion of TB patients during the treatment period. In this study, decision tree (DT), random forest (RF), support vector machine (SVM) and naïve bayes (NB) models were validated with high performance by achieving an area under the curve (AUC) of receiver operating characteristic (ROC) greater than 80%. The salient finding of the study is that the DT model was produced as a better algorithm with the highest accuracy (92.72%), an AUC (0.909), precision (95.90%), recall (95.60%) and F1-score (95.75%) among the ML models. This methodology may be used to study the precise ML model classification for predicting the treatment success of TB patients during the treatment period.

## 1. Introduction

One of the most common communicable disease in the world, tuberculosis (TB) is a major cause of death and a major contributor to poor health. Globally, the prevalence of TB was estimated at 10.6 million cases in 2022 [1]. It is estimated that 81% of TB-related deaths among HIV-negative people occurred in Africa and Southeast Asia in 2022; 29% of these deaths occurred in India alone [2]. The detection of TB should be done early and accurately to stop the transmission of the disease [3]. Artificial intelligence (AI) has the potential to speed up diagnostic processes and assist physicians in making diagnoses [4]. ML algorithms are a study of AI where statistical algorithms are used to learn or uncover underlying patterns embedded in the data [5]. In order to anticipate future data, ML “learns” a model using historical data [6]. In recent years, ML has expanded rapidly in the medical field to provide and enhance valid clinical decisions from large datasets. This is done through an algorithm that predicts an outcome, also known as a predictive model or estimator [7, 8]. Patient outcomes such as the progression of the disease and the response to a particular medication may be predicted by ML models.

The widely applied supervised ML algorithms are Logistic Regression (LR), Decision Tree (DT), Random Forest (RF), Support Vector Machine (SVM), K-Nearest Neighbor (KNN), Naïve Bayes (NB) and Neural Network (NN) [9, 10]. The complexity of the task, the nature of the data, the existence of interactions, and non-linearity all impact how well ML algorithms perform. Experimentation and model selection based on empirical performance on validation data are common practices in ML. The challenges with non-linear connections are better suited for algorithms like DT, kernelized approaches (SVM with non-linear kernels) and NN. To enhance overall performance, ensemble techniques like RF models are employed. These techniques frequently outperform individual models and offer a means of making use of the advantages of multiple algorithms [10, 11].

Few studies provide a comparative analysis of different ML algorithms to determine the most effective for TB treatment outcome prediction [13–16]. The existing studies are based on datasets from other regions, creating a research gap as their findings are less applicable to the Indian context with its unique socio-economic and healthcare dynamics of TB. With this background, we applied ML methods such as DT, RF, SVM, NB [17] in this study to predict outcome of the TB treatment for patients with TB who were registered under a clinical trial conducted by the ICMR-National Institute for Research in Tuberculosis. DT are useful because they are simple to understand, and splits data into subsets based on features but they are susceptible to overfitting depends on the nature of the data. RF is an ensemble method of multiple decision trees, reduces overfitting, handles missing values, computationally intensive and less interpretable. SVM are successful in high-dimensional areas, but their computational complexity might be a major disadvantage. NB is a probabilistic classifier assuming feature independence, simple, fast, effective for large datasets. By focusing on a dataset from an Indian clinical trial, this study addresses a significant research gap and provides insights that are more relevant to the unique healthcare dynamics of TB. The objective of the current study is to develop the best ML algorithm to evaluate TB treatment success under clinical trials using various models such as RF, SVM, NB and DT where the ML algorithm with the highest accuracy and predictive power is selected.

## 2. Materials and methods

### 2.1 Study design

A retrospective clinical trial study was conducted to estimate success rate of TB treatment using the supervised machine learning models. The STARD checklist and international guidelines for the development of ML models were followed in this study [18, 19].

### 2.2 Description of TB data used for ML analysis

A total of 1236 PTB patients enrolled in a randomized controlled trial (RCT) conducted by the ICMR ‐ National Institute for Research in Tuberculosis, Chennai, India formed the database for the current study [20]. The patients were randomly assigned to one of the three regimens which were (a) *2RE*_*3*_*HZ*_*3*_*(alt)/4RH*_*2*_ (split I): For the first two months, patients received rifampicin and ethambutol one day, isoniazid and pyrazinamide the next day, and then isoniazid plus rifampicin twice a week for the remaining four months, (b) *3RE*_*3*_*HZ*_*3*_*(alt)/3RH*_*2*_ (split II): similar to regimen (a), with each phase lasting three months, or (c) *2REHZ*_*3*_*/4RH*_*2*_ (control): rifampicin, isoniazid, ethambutol, and pyrazinamide were given three times a week for two months, then twice a week for four months for isoniazid and rifampicin. The study had gotten the necessary approval from the institutional ethics committees of ICMR-NIRT. The written informed consent had got from the patients aged greater than or equal to 18 years and the written informed assent was obtained from the parents or guardian of the patients aged below 18 years to participate in the trial. The detailed study design, methodology and other details can be found elsewhere [20]. In this study, the variables including the patients’ age in years, gender, regimen, drug susceptibility test before starting treatment, weight in kilograms after six months of treatment, and sputum culture conversion were considered for the data analysis. Our response variable for this study is sputum culture conversion which is an important predictor to evaluate during and at the end of the TB treatment period.

### 2.3 ML method

In the current study, the four popular ML techniques applied were DT [21], RF [22], SVM [23, 24] and NB [25]. The selection of an algorithm is based on the details of the given problem and the attributes of the readily available cleaned data. These various kinds of supervised learning tasks address an extensive variety of real-world challenges. The confusion matrix is used by ML classification based problems for measuring the performance of the model. It is a representation of a table that consists of estimated values and actual values of the data set. The confusion matrix is employed to evaluate metrics such as true positives (TP), false positives (FP), true negatives (TN) and false negatives (FN) as well as the accuracy of classification techniques [26–30]. The confusion matrix is made up of the elements given in Table 1.

**Table 1:** Confusion matrix.

		Predicted	
		Positive	Negative
**Actual**	Positive	TP	FP
	Negative	FN	TN

### 2.4 Data partitioning

The dataset was initially partitioned into training and testing data initially in a 70:30 ratio. The ML algorithm used the training set to determine which label the target belongs to. The prediction model was built and applied to predict the labels for the test data. The accuracy of the model was confirmed using the test data set and when the results were unsatisfactory, the learning process was continued [31]. The dataset is divided into k-folds of equal size in k-fold cross-validation which is the most popular type of cross-validation [32]. In this study, a 10-fold cross validation was applied to prevent model overfitting and to evaluate the four different ML classifiers.

The analyses of the ML algorithms described in this paper were conducted using the software RStudio 2023.06.1+524 "Mountain Hydrangea" [33]. Generally, there are different types of hyperparameters in each ML algorithm that must be tuned to enhance the results. The hyperparameters are customizable points in the software that let a machine-learning model be modified for a specific work or dataset. The hyperparameters chosen for this analysis are given in Table 2.

**Table 2:** Machine learning hyperparameters used for this study.

Model algorithms	Adjust parameter	Final parameter
**C5.0**	trails (Number of loop iterations)	1
	winnow (Whether features are filtered)	FALSE
	tree size (sizes in the case of boosting)	3
**Random Forest**	mtry (Default number of hyperparameters)	2
	n_tree (Number of tree models)	500
**Naïve Bayes**	fl (Laplace Correction)	0
	usekernel (normal density F or kernel density T)	TRUE
	adjust (Bandwidth adjustment)	1
**Support Vector** **Machines**	gamma (kernel function coefficient)	0.1
	SVM–Kernel	Radial
	Cost	2

Classification models effectively handle such categorical variable enabling accurate prediction of which specific clinical outcome will occur based on the input features. This approach is essential for making informed clinical decisions and tailoring patient care strategies. Classification models provide specific assessment metrics that are appropriate for categorical outcomes, such as accuracy, precision, recall, F1-score, ROC-AUC, and confusion matrix. In order to assess and compare the performances of all predictive models, the accuracy, recall (sensitivity), precision and F-measure (F1-score) were calculated. The F-measure computes the number of times a model made a correct prediction across the entire dataset. Furthermore, the receiver operating characteristic (ROC) area under curve (AUC) was computed to evaluate the performance of models. If the area under curve (AUC) value is higher, the classification model is better.

## 3. Results

**Fig 1:**
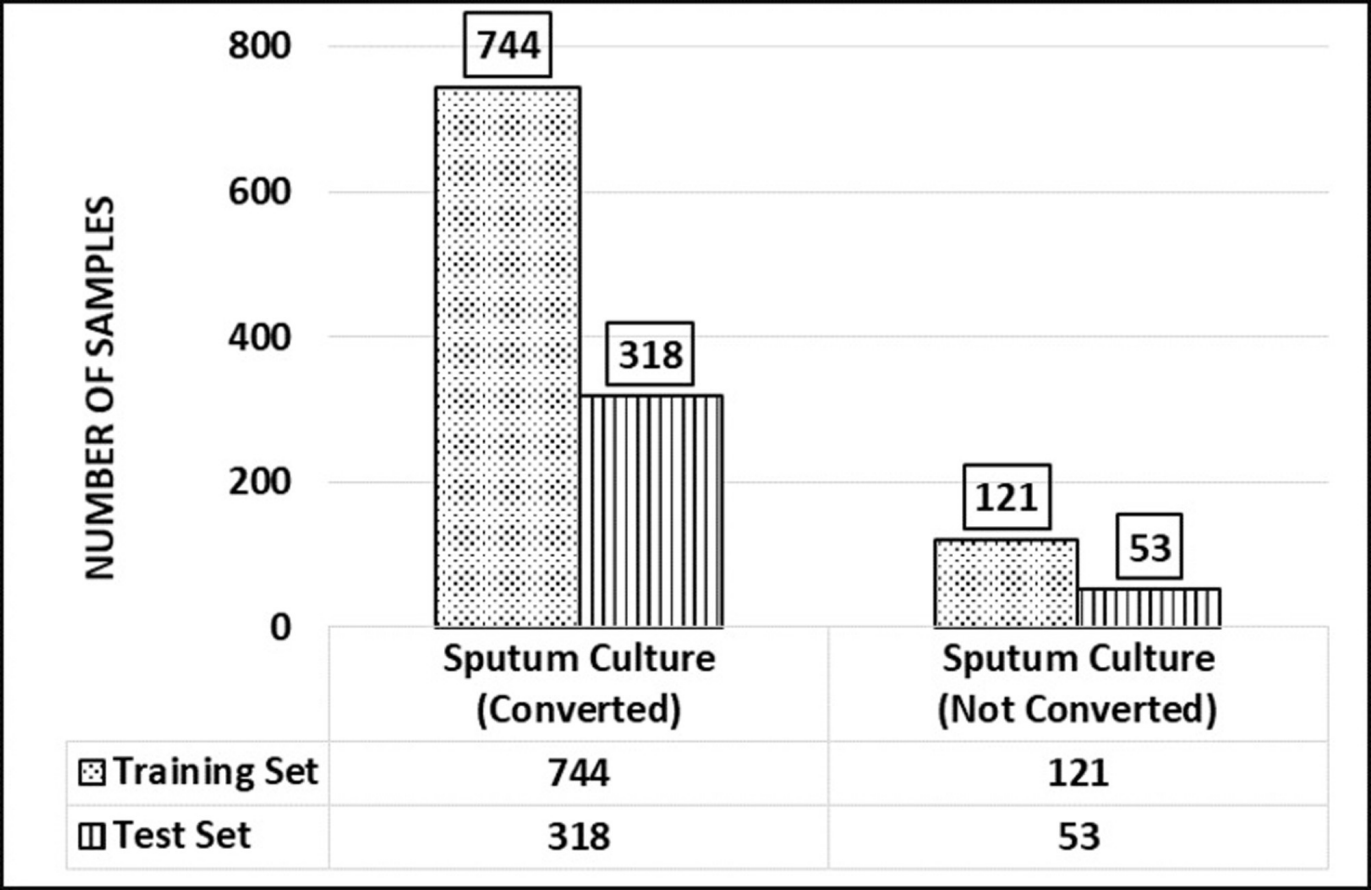
Culture conversion of TB patients for training and testing data set.

Of 1236 TB patients, 923 (74.7%) were male and 313 (25.3%) were female. The mean age of the patients was 32.9 years. The mean weight of the patients after the sixth month of the treatment period was 44.2 kilograms. Out of 1236, 1062 (85.9%) TB patients were converted to culture-negative. Of 1236, 407 (32.9%) received the regimen Split I; 415 (33.6%) were given Split II and 414 (33.5%) received the control regimen; 1006 (81.4%) were sensitive to all drugs and 230 (18.6%) were resistant to any one culture (Table 3). Fig 1 shows the treatment success of TB patients for training and testing datasets. Of 1236, the ML models were trained with 744 (70%) observations and 318 (30%) were used to test the trained ML models.

**Table 3:** Baseline characteristics of TB patients.

Variables	n (%) or mean ± SD^#^		
	Converted	Not Converted	Total
	(N = 1062)	(N = 174)	(N = 1236)
**Age**	32.8 ± 11.6	33.4 ± 10.4	32.9 ± 11.5
**Weight (in kilo gram)**	44.9 ± 6.89	40.0 ± 4.56	44.2 ± 6.82
**Sex**			
Female	281 (26.5%)	32 (18.4%)	313 (25.3%)
Male	781 (73.5%)	142 (81.6%)	923 (74.7%)
**Regimens**			
Split I	355 (33.4%)	52 (29.9%)	407 (32.9%)
Split II	369 (34.7%)	46 (26.4%)	415 (33.6%)
Control	338 (31.8%)	76 (43.7%)	414 (33.5%)
**Pre ‐ Treatment DST**			
Sensitive to all drugs	916 (86.3%)	90 (51.7%)	1006 (81.4%)
Resistant to any one drug	146 (13.7%)	84 (48.3%)	230 (18.6%)

Converted: sputum culture conversion is negative; Not converted: sputum culture conversion is positive;

^#^SD: standard deviation

The confusion matrix of the test data set for DT, RF, SVM and NB is reported in Table 4. The NB algorithm predicts the highest true positives (317 among the 371 test observations). Additionally, the SVM predicts the second highest true positives (312 out of 371 test observations) and the lowest true negatives were predicted by the NB algorithm (4 among 371 test observations).

**Table 4:** Confusion matrix of different ML algorithms for the test set.

ML Algorithms	True Positive	False Positive	True Negative	False Negative
Decision Tree	304	13	40	14
Random Forest	304	15	38	14
Support Vector Machines	312	44	9	6
Naïve Bayes	317	49	4	1

The calculated performance metrics of different ML algorithms for training and testing data sets are given in Table 5. For the training set, the RF algorithm achieved the highest precision (96.78%), accuracy (94.68%) and F1-score (96.91%) as compared to the other three ML algorithms. The DT algorithm obtained the second highest precision (95.05%), accuracy (91.91%) and F1 score (95.31%). The NB algorithm got the lowest score in comparison with DT, RF and SVM.

**Table 5:** Performance evaluation of different ML algorithms.

ML Algorithms	Performance evaluation of Training dataset				Performance evaluation of Testing dataset			
	Accuracy	Recall	Precision	F1-Score	Accuracy	Recall	Precision	F1-Score
Decision Tree	91.91%	95.56%	95.05%	95.31%	92.72%	95.60%	95.90%	95.75%
Random Forest	94.68%	97.04%	96.78%	96.91%	92.18%	95.60%	95.30%	95.45%
Support Vector Machines	88.79%	98.79%	89.31%	93.81%	86.52%	98.11%	87.64%	92.58%
Naïve Bayes	87.05%	100%	86.92%	93%	86.52%	99.69%	86.61%	92.69%

**Fig 2:**
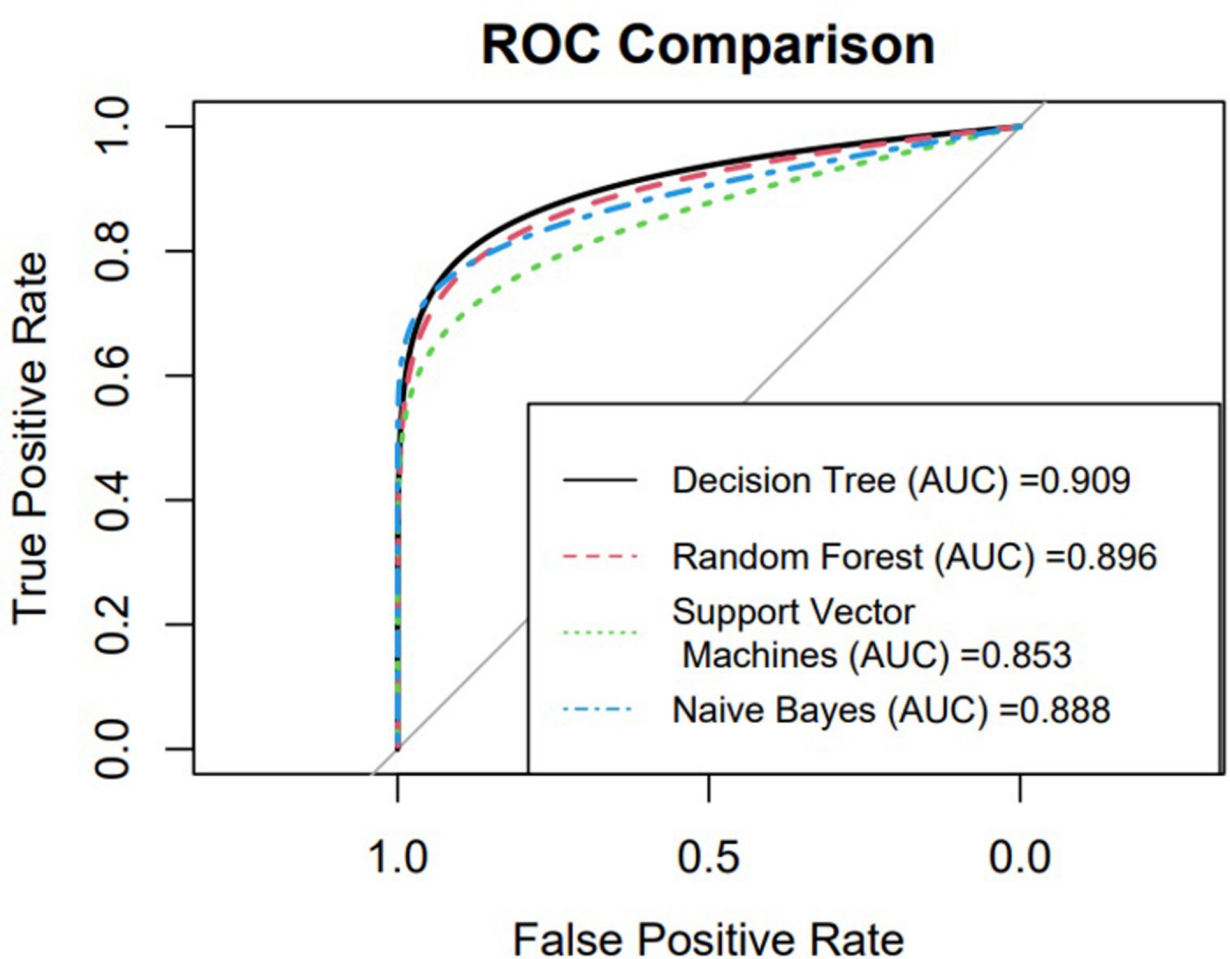
ROC curve comparison for different machine learning algorithms.

For the test set, the DT algorithm obtained the highest precision (95.90%), accuracy (92.72%) and F1 score (95.75%) as compared to the other three ML algorithms. The RF algorithm obtained the second highest precision (95.30%), accuracy (92.18%) and F1 score (95.45%). The SVM algorithm got the lowest score in comparison with DT, RF and NB. The comparison of the ROC plot for the prediction of the four ML classification models used in the study is shown in Fig 2. The highest AUC was 0.909 obtained by the DT and the second highest AUC was 0.896 obtained by the RF. Based on the results of the AUC obtained from the ROC and test dataset, the DT algorithm was performed as a good model for the data as compared to the other algorithms.

## 4. Discussion

In this study, RCT data on TB is utilized to develop and evaluate the performance of four ML algorithms (DT, RF SVM and NB) aimed at identifying sputum culture conversion among PTB patients during their treatment. Our study findings highlighted that all the four models demonstrated high performance by achieving an AUC greater than 80%. Moreover, the findings revealed that among these ML models, the DT model had achieved the highest values of metrices.

The current study estimated that around 86% of PTB patients were sputum culture negative at the end of treatment. Numerous studies reported that early sputum culture conversion is a vital factor for treatment success in susceptible and drug resistant TB [34–37]. Our study findings corroborated with a study where various ML algorithms were employed and the DT method was determined to be the best algorithm for predicting the outcome of TB treatment [15]. Another study with extra pulmonary TB and PTB data reported that the DT model performed well and achieved the highest level of accuracy at 95% for disease classification [38]. Asha et al, 2011 reported that SVM and DT are the best models among the basic learning classifiers compared with KNN, NB whereas RF is the best model among the ensemble classifiers compared with Bagging and Adaboost for predicting the effective classification accuracy of TB [16]. A study from Kenya recommended that ML correctly predict confirmation of TB with clinical, demographic, and radiologic factors among young children [39]. Hussain et al (2018) reported that the best model is the RF (accuracy = 76.32%) as compared to the SVM and the NN for evaluating the treatment outcome of drug-susceptible TB patients [13]. A retrospective study from Uganda discussed that the SVM (91.28%) achieved the highest accuracy among the five ML algorithms (LR, ANN, SVM, RF, and AdaBoost) to explore the risk factors for non-adherence to TB treatment [14].

Comparative evaluation and testing different algorithms on the same dataset are often essential to making an informed decision about the most suitable approach. In this study, DT produced the best algorithm for analyzing the clinical trial TB data due to the specific characteristics of the dataset and the goals of the analyses. While DT, RF, SVM and NB are effective in various settings, advanced methods like Gradient Boosting Machines, Deep Learning (NN), Deep Belief Network (DBN) and advanced Ensemble Methods offer enhanced capabilities in terms of accuracy, handling complex relationships, and scalability to large datasets [12, 40]. A DBN survival Cox model was compared to predict the overall survival in Osteosarcoma patients with the other ML algorithms and identified that this DNB algorithm demonstrated better performance [41]. A study from china documented that the performance of the RF model ranked best among the six ML prediction models to validate a risk prediction model for lymph node metastasis of Ewing’s sarcoma [42].

The choice of method often depends on the specific characteristics of the data, computational resources, and interpretability requirements in clinical applications such as predicting TB treatment outcomes [43]. The methodology used in the study is robust predictive ML algorithms specifically in the context of TB data, emphasizing their potential utility in clinical settings for predicting treatment outcomes related to culture conversion.

The data utilized in this article were limited and did not include other important valuations of TB diagnosis related to cure or treatment outcome. Even though this study was focused on a small number of attributes, we were still able to utilize the right classifier and optimize hyperparameters to reach an impressive result. In the current study, extra PTB and multidrug resistant TB data were not considered. The performance and consistency of the models vary across clinical applications, and depend on the quality and completeness of the disease data.

## 5. Conclusion

In this study, we explored and tested four ML algorithms for predicting sputum culture conversion during TB treatment in clinical trial settings. Our study findings support the utility of the ML algorithms to predict treatment success for patients with TB. This study concluded that DT is a valid tool with superior performance to predict sputum culture conversion, which determines the success or failure of TB treatment. The study has offered methodological insights into the application of these ML algorithms in the healthcare domain, which can be valuable for future research and implementation in similar settings. The implementation of these models in clinical settings has the potential to improve decision-making for doctors involved in treating TB. The models provide predictive insights that enable personalized and proactive care strategies, ultimately improving treatment outcomes and patient management in TB healthcare settings. Future research focusing on specific aspects of TB diagnosis that are connected to the conversion of sputum cultures might provide a greater understanding of the disease’s recovery process. In addition, the use of boosting algorithms may result in higher accuracy than that of simple classification algorithms.

## Supporting information

S1 File This is a minimal dataset for this analysis. (PNG)
