# Supplementary figures and images for: Machine learning algorithms to predict treatment success for patients with pulmonary tuberculosis
Source: PLoS One. 2024 Oct 16;19(10):e0309151. doi: 10.1371/journal.pone.0309151 (PMC11482692; doi:10.1371/journal.pone.0309151)

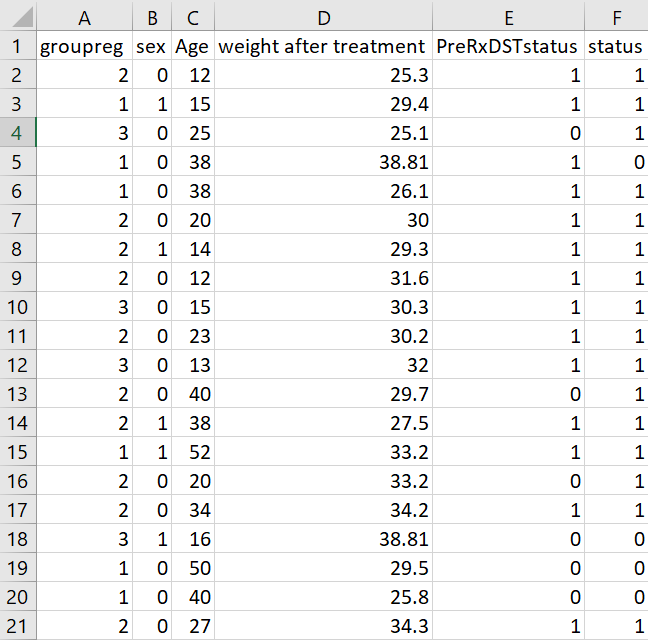

Supplement: S1 File — (PNG) [file pone.0309151.s001.png]
